# Apoptotic cell death induced by copper (II), manganese (II) and silver (I) complexes containing bridging dicarboxylate and 1,10-phenanthroline ligands: one of the multi-modes of anticancer activity?

**DOI:** 10.1007/s10534-025-00676-8

**Published:** 2025-03-17

**Authors:** Ella O’Sullivan, Denis O’Shea, Michael Devereux, Orla Howe

**Affiliations:** 1 School of Biological, Health and Sports Sciences, Technological University Dublin, Dublin, Ireland; 2 Sustainability and Health Research Hub, Technological University Dublin, Dublin, Ireland; 3 School of Food Science and Environmental Health, Technological University Dublin, Dublin, Ireland; 4 The Physical to Life Sciences Research Hub, Technological University Dublin, Dublin, Ireland

**Keywords:** Metal complexes, 1,10-Phenanthroline, Anti-cancer, ROS production, Apoptosis

## Abstract

Cu(II), Mn(II) and Ag(I) complexes incorporating bridging dicarboxylate and 1,10-phenanthroline ligands have exhibited anti-cancer potential with significant in vitro and in vivo cytotoxic efficacies. Our study focuses on regulated cell death process of apoptosis as a mode of action of the anti-cancer activity by the complexes. Cytotoxicity screening of the complexes demonstrated all the metal-dicarboxylate-phenanthroline complexes exhibit superior activity compared to their non-phenanthroline containing precursors, in addition to cisplatin. The Cu(II) and Mn(II) complexes were shown to induce reactive oxygen species (ROS) but this was not observed for the Ag(I) analogues. Furthermore, apoptosis was found to be induced by all the metal-phenanthroline complexes to varying degrees contingent on the type of metal centre in the complex. Apoptotic gene expression analysis established the predominant activation of the intrinsic apoptotic pathway, with co-stimulation of the extrinsic pathway observed in some cases. The mechanistic data provided within this study highlights the multi-modal activity of the metal-phenanthroline complexes contingent on the type of metal present, warranting continued investigation of their biological modes of action beyond apoptosis induction.

**Supplementary Information:**

The online version contains supplementary material available at 10.1007/s10534-025-00676-8.

## Introduction

Transition metal complexes with 1,10-phenathroline have previously demonstrated anti-microbial, (Viganor et al. 2016; McCarron et al. 2018; Ahmed et al. 2019; Ventura et al. 2020; Vianez Peregrino et al. 2021) anti-fungal, (Santos et al., 2012; Gandra et al. 2017; Gandra et al. 2020; Granato et al. 2017; Granato et al. 2021) and anti-cancer, (Kellett et al. 2012; Thornton et al. 2016; Rochford et al. 2018; Rochford et al. 2020) activity. 1,10-Phenanthroline is a bioactive chelate commonly used in coordination chemistry (Đurić et al. 2020; Nunes et al. 2020). The structure of the aromatic *N, N*-ligand allows for it to complex with metal ions quickly. Its planar nature has been shown to facilitate the ligands interaction with DNA and RNA, through both intercalating and groove-binding interactions and therefore the potential to interact with nucleic acids and alter molecular processes in cells (Sammes and Yahioglu 1994; Accorsi et al. 2009; Alreja and Kaur 2016).

There have been numerous efforts by our group and collaborators to understand the multi-modal mechanisms of action of the metal-phen complexes in various biological systems. A series of bimetallic Cu(II) and Mn(II) complexes incorporating chelating 1,10-phenanthroline and bridging octanedioate ligands demonstrated impressive cytotoxicity towards an array of cell lines (MCF-7, DU145, HT29, and SK-OV-3), eliciting their effects through the generation of reactive oxygen species (ROS), incurring DNA damage and affecting superoxide dismutase (SOD) activity. Several of these complexes have exhibited impressive nano- and pico-molar cytotoxicity towards cancer cell lines, with reduced toxicity observed towards non-cancerous cells. They were also shown to be well tolerated in vivo using *Galleria Mellonella* models when compared to cisplatin (Kellett et al. 2011).

A series of Cu(II)-phenanthroline complexes were characterised using both in vitro and in vivo models for activity and mechanistic profiles. Rochford et al. (Rochford et al. 2018; Rochford et al. 2020) found that the copper-phenanthroline-phenazine complexes [Cu(phen)_2_]^2+^, [Cu(DPQ)(Phen)]^2+^, and [Cu(DPPZ)(Phen)]^2+^ induce apoptosis via the mitochondrial pathway and that the mitochondrial function is integral to the response in vitro (Rochford et al. 2020). The same complexes were toxic but did not induce an immune response in vivo in *Galleria Mellonella*. This toxicity was attributed to complex proteomic profiles in various pathways such as purine synthesis, glycolysis and other detoxification and metabolic processes (Rochford et al. 2018). Furthermore, Mn(II) complexes present as interesting candidates for cancer therapy. The dinuclear cationic Mn complex [Mn_2_(μ-oda)(phen)_4_(H_2_O)_2_]^2+^ has been reported to be a potent inducer of reactive oxygen species, with this activity being one of its primary mechanisms for inducing cancer cell death (Kellett et al. 2011). Similar to its Cu(II) analogue, this complex acts as a superoxide dismutase mimetic and exhibits DNA-binding abilities. However, unlike the Cu(II) analogue, self-cleaving activity has not been reported for this complex. Instead, it has been theorised that its action occurs indirectly through ROS production rather than direct DNA damage. The complex demonstrated nano-molar cytotoxicity towards a series of colorectal cancers (HT29, SW480 and SW620) with an almost tenfold reduction in potency towards non-cancerous (HaCaT) cells. In addition, it was found to be well-tolerated in vivo by *Galleria Mellonella* (Kellett et al. 2011; Slator et al. 2017). Furthermore, Slator et al. 2017 (Slator et al. 2017) reported the ability of the complex to induce autophagy, which preceded the subsequent activation of the intrinsic-apoptotic pathway, acting as the executioner of cell death. The findings in this study comprehensively illustrated the intricate crosstalk between cellular mechanisms, demonstrating the ability of the manganese complex to induce cell death by leveraging various biological systems.

Herein we present biological activity profiles of a series of copper (II), manganese (II) and silver (I) complexes, incorporating chelating 1,10-phenathroline and bridging dicarboxylate ligands, towards breast cancer and breast normal cells in vitro. Our objective was to understand how these different metal complexes exhibit their cytotoxic activity in these cells. Apoptosis is a classic regulated cell death (RCD) pathway with its molecular mechanisms well known. It is well-documented to be an induced RCD pathway by various toxic agents including therapeutic drugs that lead to cytotoxicity and has been reported as a mode of action for a variety of metal-based drugs, particularly, Cu(II) complexes (Gałczyńska et al., 2020; Molinaro et al. 2020). Our study incorporates a gene expression analysis in which several of the key genes that signal the apoptotic cascade were investigated. Apoptosis is divided into two distinct pathways, the intrinsic and extrinsic pathway, with an extensive range of proteins involved in the progression and execution of both. During the intrinsic cascade, pro-apoptotic members of the B cell lymphoma 2 family such as BCL-2 associated x protein and BCL-2 homologous antagonist killer (*BAX/BAK*) act as monomers leading to the formation of pores on the mitochondrial membrane that cause mitochondrial outer membrane permeabilization (MOMP) (Galluzzi et al. 2018). This facilitates the release of cytochrome c from the mitochondria which complexes with Apaf-1 (apoptotic protease activating factor-1) and pro-caspase 9 in the formation of a apoptosome complex, with Caspase-9 cleaving and thereby activating *caspase-3 or -7* to execute the final stages of apoptotic cell death. Caspases are serine proteases central to the signalling of Apoptosis with initiator Caspases,(*caspase-9 and Caspase-8)* required for the initiation of the intrinsic and extrinsic apoptotic pathway respectively and the execution of both pathways through executioner Caspases (Caspase-3 or -7). After MOMP in the intrinsic pathway, cytochrome c release is accompanied by the release of the inhibitors serine protease *HtrA2/OMI* and second mitochondria-derived activator of caspases (*SMAC*). These proteins suppress the activity of apoptotic inhibitors like X-linked inhibitor of apoptosis (*XIAP*) which obstruct the apoptotic pathway by inhibiting the activity of caspases. BH3-only proteins (*BIM/BID/BAD*) play a crucial role to switch-on the apoptotic signalling cascade, by activating pro-apoptotic members of the BCL-2 family thus enabling the progression of the above cascade (Baig et al. 2016; Tang et al. 2019; Al-Aamri et al. 2021).

The stimulation of the extrinsic, or ‘death receptor’ apoptotic pathway relies on the binding of specific ligands to cell surface receptors, known as ‘death receptors’, belonging to the tumour necrosis factor (TNF) family. One such interaction involves the binding of the Fas ligand (FasL) to its corresponding receptor, FasR (CD95), which stimulates the trimerization of the receptor, in turn recruiting the cytoplasmic adaptor protein FADD (Fas-Associated Death Domain) by forming homotypical interactions of the death domains in both FADD and FasR. The additional death effector domain (DED) of FADD facilitates the binding of procaspase-8 via its DED, forming the death inducing signalling complex (DISC). Subsequently, procaspase-8 molecules are dimerised, enabling autocatalytic cleavage and activation of caspase-8. This initiator caspase-8 proceeds to activate executioner caspases-3 and -7 to complete the cell death pathway. Caspase-8 can also stimulate the intrinsic pathway, with pro-apoptotic Bid functioning as a bridge crossing the pathways. Caspase-8 can cleave the protein, forming truncated tBid, which translocates to the mitochondria and prompts the release of cytochrome c, in turn stimulating the previously described signalling cascade involving caspase-9 (Roy and Nicholson 2000; Ocker and Höpfner 2012; Green 2022).

Reactive oxygen species, produced as by-products of cellular metabolism, are closely linked to the regulation of the apoptotic pathway. The relationship between apoptosis regulation and ROS is complex, with an array of cellular mechanisms involved. An intricate balance of ROS is necessary in normal cellular conditions, with low levels of ROS essential for functions such as cell cycle progression, differentiation and cell death. This balance in normal conditions is maintained by an antioxidant defence system such as superoxide dismutase (SOD), catalase and glutathione (GSH). Dysregulation of ROS production leads to an excess of oxidative stress, incurring damage to proteins, nucleic acids and cellular organelles, in turn inducing the apoptotic cell death pathway (Mateâ and Saâ Nchez-Jimeâ Nez 2000; Redza-Dutordoir and Averill-Bates 2016). This stress can stimulate the transcription factor p53 to downregulate pro-survival proteins such as BCL-2 and IAPs, in addition to upregulating pro-apoptotic proteins. P53 is responsible for activating the transcription of proteins both involved in the intrinsic (Bax, Bid, Puma, Apaf-1) and the extrinsic (Fas, FasL, DR-4/5) pathway. Moreover, ROS can oxidise proteins essential for cellular survival, in turn stimulating cell death via apoptosis. Additional cellular targets have been implicated in the induction of apoptosis by ROS, with the mechanisms not yet fully understood (Redza-Dutordoir and Averill-Bates 2016; Villalpando-Rodriguez and Gibson 2021).

The biological activity of a series of previously published binuclear Copper (II), Manganese (II) and Silver (I) complexes containing bridging dicarboxylate and 1,10-Phenanthroline (phen) ligands in breast cancer and normal cells in vitro are presented herein. The results of an investigation of the influence of the different metal centres on the induction of the RCD apoptotic pathway are also discussed. To gain further mechanistic insights, the induction of reactive oxygen species (ROS) by the complexes was assessed to determine a link between ROS and apoptosis induction. Gene expression studies, focused on apoptotic genes and regulators of the pathways, were also performed to determine if a specific apoptotic pathway was targeted by the different complexes and indeed to determine any specific unknown biological targets.

## Materials and methods

### Mammalian cell culture conditions and maintenance

The MCF-7 (human breast cancer) and MCF-12A (non-tumourigenic human breast) cell lines were obtained from the Technological University Dublin (TUDublin, City Campus) internal cell bank and commercially from the American Type Culture Collection (ATCC), respectively. Both cell lines were cultured using Dulbecco’s Modified Eagle Medium/Nutrient Mixture F-12 Ham (DMEM-F12) (Sigma, Ireland) (Cat# D6421) media. The MCF-7 media was supplemented with 10% Foetal Bovine Serum (Sigma, Ireland) (Cat# F7524) and 1% L-Glutamine (Sigma, Ireland) (Cat# 592020C). The MCF-12A media was supplemented with 5% Equine Serum (Sigma, Ireland) (Cat# H0146) 1.25% L-Glutamine (Sigma, Ireland) (Cat# 592020C), 20 ng/ml Human Epidermal Growth Factor (Thermo Fisher Scientific, Ireland) (Cat# PHG0313), 0.01 mg/ml insulin (Thermo Fisher Scientific, Ireland)(Cat# 12585014) and 500 ng/ml hydrocortisone (Sigma, Ireland) (Cat# H0888). The cells were grown in T75 culture flasks (Sarstedt, Germany) (Cat# 83.3911.002) and were incubated in a 5% CO_2_ atmosphere at 37 ℃. The cells were monitored daily, and once 80–90% confluence was reached, they were subcultured with an enzymatic dissociation method using trypsin. Both cell lines were routinely subcultured for 1 week prior to beginning experimental work, to ensure the cells had adapted to the environment.

### Test complexes

The complexes (**1–8**) analysed herein were synthesised by inorganic chemists at Technological University Dublin using previously published methods (Devereux et al. 1999; Mccann et al. 1995; Thornton et al. 2016). The complexes incorporated either copper(II) (Cu^2+^), silver(I) (Ag^+^) or manganese(II) (Mn^2+^) centres coordinated to 1,10- phenanthroline (phen) and either octanedioate (oda^2−^) or undecanedioate (udda^2−^) ligands. Complexes **1–8** were purified and characterised in accordance with the methods previously published. Complexes **1–8** were formulated as outlined in Table 1. Complexes **1**-**5**, **7,** and **8** were dissolved in distilled H_2_O, and complex **6** was dissolved in DMSO, solvents in which the active components of the complexes are known to be stable. The silver complex remains intact as a neutral species in solution, while the copper complex dissociates to form the di-cationic active species, and the manganese double salt dissociates to release the di-cationic species, similar to the copper active component. Complexes **4**, **5**, **7** and **8** dissolved readily in distilled H_2_O. Whereas complexes **1**, **2**, **3** and **6** required additional agitation in their respective solvents to achieve complete dissociation. Free 1,10-Phenanthroline (**Phen**) and cisplatin were also analysed as controls. **Phen** was dissolved in distilled H_2_O and cisplatin was dissolved in DMSO. Both dissolved readily.

**Table 1:** The metal complexes investigated in this study, with corresponding solvents and molecular weights

Code	Complex formula	Solvent	Molecular weight (g/mol)	Synthesis reference
Copper (II) complexes				
Complex 1	[Cu_2_(oda)(phen)_4_](ClO_4_)_2_	H_2_O	1219.03	Devereux et al. (1999)
Complex 2	[Cu(oda)(phen)_2_]	H_2_O; EtOH	595.7	McCann et al. (1995)
Complex 3	[Cu_2_(oda)_2_]	H_2_O; EtOH	471.2	McCann et al. (1995)
Silver (I) complexes				
Complex 4	[Ag_2_(oda)(phen)_3_]	H_2_O	928.58	Thornton et al. (2016)
Complex 5	[Ag_2_(udda)(phen)_3_]	H_2_O	970.64	Thornton et al. (2016)
Complex 6	[Ag_2_(oda)]	DMSO	387.94	Thornton et al. (2016)
Manganese (II) complexes				
Complex 7	{[Mn_2_(oda)_3_(phen)_4_]^2−^[Mn_2_(oda)(phen)_4_ (H_2_O)_2_]^2+^}	H_2_O	2384.28	Casey et al. (1994)
Complex 8	[Mn(oda)]⋅H_2_O	H_2_O; EtOH	245.14	Casey et al. (1994)
Controls				
Phen	1,10-Phenanthroline	H_2_O	180.21	
	Cisplatin	DMSO	300.05	

phen = 1,10-phenanthroline; oda^2−^ = octanedioate; udda^2−^ = undecanedioate

### In Vitro cytotoxicity assessment

The MCF-7 and MCF-12A cells were seeded at a density of 1 × 10^4^ in 96-well flat bottom plates (Sarstedt, Germany) (Cat# 83.3924) and incubated in a 5% CO_2_ atmosphere at 37 ℃ for 24 h prior to exposure to the complexes. Following the incubation period, the media was removed, and cells were washed with sterile phosphate-buffered saline (PBS). The cells were then exposed to complexes **1**–**8**, free 1,10-Phenanthroline and cisplatin (Sigma, Ireland) (Cat #232120). All agents were diluted in supplemented cell culture media, and the cells were exposed to a concentration range of 0.5 μM–100 μM for 24 h in the conditions outlined previously. Following the exposure period, the drug-containing media was removed, and cells were washed three times with PBS, ensuring complete removal of the test agents. A 5% working solution of Alamar Blue (Thermo Fisher Scientific, Ireland) (Cat# DAL1100) was prepared in dark conditions using unsupplemented cell culture media. 100 μl of the solution was added to each well, and the cells were incubated for 3 h. Following incubation, absorbance values were obtained using a Varioskan Lux (Thermo Scientific, USA) at 570 nm and 600 nm wavelengths. Each exposure was carried out in triplicate plates and was repeated three independent times.

### Detection of reactive oxygen species

The production of reactive oxygen species (ROS) was detected in MCF-7 and MCF12A cells using the cell-permeant dye 2',7'- dichlorodihydrofluorescein diacetate (H2DCFDA) (Thermo Fisher Scientific, Ireland) (Cat# D399). Cells were seeded at a density of 1 × 10^4^ in 96 well plates and incubated for 24 h. Post-incubation, the media was removed, and cells were washed with PBS. A 10 μM DCFH-DA working solution was prepared using cell culture media, and the cells were incubated with 100 μl of the solution in the dark for 30 min. The cells were then washed with PBS, and exposed to 4 μM H_2_O_2_ (positive control), complexes **1**,**2**,**4**,**5**,**7**, **Phen** and cisplatin at a concentration range of 0.78 μM–50 μM. Fluorescent measurements were recorded at 15 min, 30 min, 1 h, and subsequently every hour up to 6 h using a Varioskan Lux™ multimode microplate reader (Thermo Fisher Scientific, Ireland) at an excitation wavelength of 495 nm and emission of 527 nm. The assay was repeated three independent times, using three replicates per plate.

### Detection of apoptosis induction with flow cytometry

MCF-7 and MCF-12A cells were seeded at a density of 1 × 10^5^ in T25 flasks (Sarstedt, Germany) and incubated for 24 h at 37 °C with 5% CO_2_ allowing for attachment. Post -incubation, the media was removed, cells were washed with sterile PBS and then treated using IC_25_ concentrations established from the in vitro cytotoxicity screen. The cells were exposed to complexes **1**,**2**,**4**,**5**,**6**,**7**, **Phen** and cisplatin for 24 h. After the exposure period, the cells were enzymatically detached using trypsin, collected and centrifuged at 1200RPM for 10 min. The cells were then washed with PBS, re-centrifuged and resuspended in 1 ml of annexin binding buffer. Prior to staining, the concentration of each sample was adjusted to 1 × 10^6^ cells/ml. The cells were stained with 1 μl of propidium iodide (PI) (Sigma, Ireland) (Cat #P4864) and 2 μl of Annexin V (Thermo Fisher Scientific, Ireland) (Cat #A13199). The cells were incubated at room temperature for 15 min prior to analysis. The staining process was completed in the dark to eliminate interference with photo-sensitive reagents. Immediately prior to analysis by flow cytometry, 400 μl of annexin binding buffer was added to each sample. Samples were analysed using a (CytoFLEX) (Beckman Coulter, USA). The AV/PI assay was completed three independent times.

### Detection of caspase 3/7 expression with flow cytometry

The expression of caspase-3/7 in MCF-7 and MCF-12A cells was determined using the VybrantTM FAM™ Caspase-3 and Caspase-7 Assay Kit (Cat# V35118) (Thermo Fisher Scientific, Ireland). Cells were seeded at a density of 1 × 10^5^ in T25 flasks and incubated in routine conditions for 24 h. The media was removed, the cells were washed with PBS and were then exposed to complexes **2**, **4** and **7** and cisplatin at IC_25_ concentrations for 24 h. Post-exposure, the cells were harvested for staining.

### Apoptosis-related gene expression

A broad scope of molecular targets involved in the apoptotic pathway were selected for analysis using qRT-PCR including initiators, mediators and inhibitors. These gene targets along with their cellular location and associated primer set are outlined in Table 2. Primer sets for each gene were designed using NCBI nucleotide sequences and the Primer3 application (Table 3). Once designed, the primers were synthesised by Sigma Aldrich, Ireland.

**Table 2:** Target genes, their function, and cellular locations

Gene	Function	Cellular location
Caspase-9	Apoptosis initiator	Cytoplasm
Caspase-8	Apoptosis initiator	
Caspase-3	Apoptosis executioner	
BAD	Pro-apoptotic mediator in intrinsic pathway	
BAX	Pro- and anti-apoptotic mediator	
BCL2	Pro- and anti-apoptotic mediator	
BIM	Pro-apoptotic mediator in intrinsic pathway	
BID		
XIAP	Inhibits apoptosis (negative regulation)	
IAP		
IAP-2		
SMAC (DIABLO)	Promotes apoptosis by inhibiting IAPs	Mitochondria
OMI (HTRA2)		
Actin	Contractile microfilaments for movement	Cytoplasm (Reference genes)
β-Tubulin	Polymerizes into microtubules for growth	Cytoplasm (Reference genes)

**Table 3:** Target genes and their associated oligonucleotides

Cellular location	Gene	Oligonucleotide sequences (5’-3’)
Cytoplasm	Caspase-9	AAA GTT GTC GAA GCC AAC CCC (F) GAC TCA CGG CAG AAG TTC AC (R)
	Caspase-8	TGC CCA AAT CAA ACA GAG GCC (F) ACA GAT ACC CCC GAG GTT GC (R)
	Caspase-3	AGA TGT CGA TGC GAC GAA ACC (F) GCA CAC AAA CAA AAA CTC GTC C (R)
	BAD	TGG TGG GAT CGG AAC TTG G (F) AAA GGA GAC GCA CGA CAT CC (R)
	BAX	GGC CTA CCC TAG ACA CAT GG (F) TTC TGC TAA GCT CTC CAC G (R)
	BCL2	TTG AGC AGC TTT GGA ACC (F) CCG ATT GAT GAT GCC CTT GG (R)
	BIM	CTT TCT GGC CCT TGT TCC C (F) TTG TGG CTC TGT GGA TGG (R)
	BID	GGC CTA CCC TAG ACA CAT GG (F) TTC TGC TAA GCT CTC CAC G (R)
	XIAP	GTG TTG AAT GGG GAA AGG GG (F) ATC TCC TGA CTC GTG ATC C (R)
	IAP	GTT GTG TAG TTG GGC TTG AGG (F) TGG GTC AGC AAT TTT CTC CC (R)
	IAP-2	ATG TGG GAC TCA GGT GTT GG (F) TGC ATT TTC ATC TCC TGG GC (R)
Mitochondria	SMAC (DIABLO)	AGC TGC ATA TCA AAC TGG CGG (F) CCT GTG TTT TTC TGA CGG AGC (R)
	OMI (HTRA2)	CAG AAC ACG ATA CAC TCC GG (F) TTC ACT CCA ATC ACC TCC CC (R)
Cytoplasm (Reference genes)	Actin	ACT CTT CCA GCC TTC TTC C (F) GTT GGC GTA CAG GTC TTG GC (R)
Cytoplasm (Reference genes)	β-Tubulin	GCT TCT TGG TTT CCA CAG C (F) CGT TCT CAG GTC TGC ACC TC (R)

‘F’ refers to forward primer, ‘R’ refers to reverse primer

MCF-7 and MCF-12A cells were seeded at a density of 1 × 10^5^ in T25 flasks and incubated for 24 h allowing for attachment. Following incubation, the seeding media was removed, and the cells were washed with PBS. The cells were then exposed to complexes **1**,**2**,**4**,**5**,**6**,**7**, **Phen** and cisplatin for 24 h using IC_25_ concentrations. Post-exposure, RNA was isolated from the cells. Tri-reagent^Ⓡ^ (Sigma, Ireland) (Cat# T9424) was added to the cells followed by centrifugation at 300 g for 5 min. The RNA was precipitated using chloroform, followed by centrifugation and washing of the pellet using 75% ethanol. The RNA was resuspended in 30 μl of nuclease-free water. The RNA was either used immediately for qRT-PCR or stored at −80 ℃. The RNA from each sample was quantified using the NanoDrop™ 2000 Spectrophotometer (Thermo Fisher Scientific, Ireland). The A260/A230 ratio was measured to ensure the purity of each sample. Prior to cDNA synthesis, all samples were adjusted to a concentration of 100 ng. The RNA was then reverse transcribed into cDNA using the Quanta Biosciences Kit (Quanta Biosciences, USA) (Cat# 95047). Using the SimpliAmp Thermal Cycler (Applied Biosystems; Thermo Fisher Scientific, Ireland), the following settings were applied: 5 min at 22 ℃, 30 min at 42 ℃, 5 min at 85 ℃; samples were then maintained in an indefinite hold at 4 ℃ prior to being used for quantitative real-time PCR. The cDNA templates were amplified and analysed using SYBR™ Green (Fisher Scientific, UK) (Cat#10187094). Using the 7500 fast Real-Time PCR System (Applied Biosystems, USA), the following thermal cycling setting were applied for amplification: Pre-Incubation (1 cycle) 95 ℃, 5 Minutes, 4.4 ℃ ramp; Amplification (45 cycles) 95 °C 10 s ramp 4.4 °C, 60 °C 10 s ramp 2.2 °C, 72 °C 10 s ramp 4.4 °C; Melting Curve (1 cycle) 95 °C 5 s ramp 4.4 °C, 65 °C 1 min ramp 2.2 °C, 97 °C continuous ramp 0.11 °C acquisition 10 per °C; Cooling (1 cycle) 40 °C 10 s ramp 1.5 °C. Two reference genes, actin and tubulin, were employed to determine the expression of the target genes. No template controls were included to confirm the absence of template contamination and auto-fluorescence. Each PCR plate contained three technical replicates per sample and gene target, with each experiment repeated three independent times using three individual biological replicates.

### Statistical analysis

The software packages Microsoft^Ⓡ^ Excel (USA) and GraphPad Prism (Ver. 10.0.3) (GraphPad, USA) were used to analyse the alamar blue data. The treated sample values were first normalised to the negative control values. After normalisation, the mean and standard deviations (SD) were established using Microsoft^Ⓡ^ Excel. The statistical analysis package GraphPad was then used to determine the IC_25_ and IC_50_ concentrations. IC_25_ and IC_50_ values represent the test drug concentration at which there is a 25% and 50% reduction in cell viability, respectively. The IC_25_ concentrations were chosen for subsequent experimental work due to the high cytotoxic capacity of the complexes. The efficacy of the test complexes was compared with that of cisplatin against each cell line using a one-way ANOVA with Tukey’s Post Hoc test to determine if there was a statistically significant difference between the treatments. A *p* value of 0.05 or lower is considered to be statistically significant.

Flow cytometry data analysis was performed with the CytExpert package (Ver. 2.4) (Beckman Coulter, USA) and Microsoft^Ⓡ^ Excel (USA). The mean MFI and percentage cells were calculated using these packages, followed by one-way ANOVA analysis with post hoc Bonferroni correction using GraphPad Prism (Ver. 9.4) (GraphPad, USA).

qRT-PCR data was analysed by first using the Vandesompele method to calculate the geometric mean of Ct values for the reference genes, actin and tubulin. These values were used for normalisation of the target gene expression values which were then calculated using the Livak and Schmittgen formula: 2^−ΔCt1(test)−ΔCt2(control)^ (Livak and Schmittgen 2001; Vandesompele et al. 2002).

## Results

### In Vitro cytotoxicity assessment

The cytotoxicity of complexes **1**–**8**, free 1,10-phenanthroline and cisplatin was established in both MCF-7 and MCF-12A cell lines (Tables 4 and 5) using the alamar blue assay. This assay was applied subsequent to 24-h exposure to the test complexes at a concentration range of 0.5 μM–100 μM.

**Table 4:** IC_25_ values for MCF-7 and MCF-12A cells following 24-h exposure to metal-phenanthroline complexes, 1,10-phenanthroline and cisplatin

	Cytotoxic activity IC_25_ (μM) ± SD	
Complex	MCF-7	MCF-12A
[Cu_2_(oda)(phen)_4_](ClO_4_)_2_] (**1**)	1.77 ± 0.14**	4.86 ± 1.91
[Cu(oda)(phen)_2_] (**2**)	2.04 ± 0.24*	0.68 ± 0.13
[Cu_2_(oda)_2_] (**3**)	> 100	> 100
[Ag_2_(oda)(phen)_3_] (**4**)	5.52 ± 0.89	4.91 ± 0.14
[Ag_2_(udda)(phen)_3_] (**5**)	5.84 ± 0.12	5.52 ± 0.51
[Ag_2_(oda)] (**6**)	13.90 ± 2.93	6.43 ± 4.9
{[Mn_2_(oda)_3_(phen)_4_]^2−^[Mn_2_(oda)(phen)_4_ (H_2_O)_2_]^2+^} (**7**)	0.70 ± 0.11**	0.38 ± 0.39
[Mn(oda)]⋅H_2_O (**8**)	> 100	> 100
1,10-phenathroline	9.02 ± 4.26	11.14 ± 4.39
Cisplatin	11.77 ± 6.6	7.84 ± 2.47

IC_25_ (μM) represents the concentration at which there is a 25% reduction in metabolic activity in treated cells compared to untreated control cells. Mean ± SD (N = 3). * denotes *p* value < 0.05 compared to cisplatin; ** denotes *p* value < 0.01 compared to cisplatin; ***p value < 0.001 compared to cisplatin; ****p value < 0.0001 compared to cisplatin

**Table 5:** IC_50_ values for MCF-7 and MCF-12A cells following 24-h exposure to metal-phenanthroline complexes, 1,10-phenanthroline and cisplatin

	Cytotoxic activity IC_50_ (μM) ± SD	
Complex	MCF-7	MCF-12A
[Cu_2_(oda)(phen)_4_](ClO_4_)_2_] (**1**)	4.76 ± 0.30****	6.14 ± 0.37*
[Cu(oda)(phen)_2_] (**2**)	6.72 ± 0.60****	1.65 ± 0.13**
[Cu_2_(oda)_2_] (**3**)	> 100	> 100
[Ag_2_(oda)(phen)_3_] (**4**)	13.53 ± 1.48****	6.12 ± 0.33*
[Ag_2_(udda)(phen)_3_] (**5**)	7.68 ± 0.09****	6.78 ± 0.50*
[Ag_2_(oda)] (**6**)	26.77 ± 7.14*	14.36 ± 3.12
{[Mn_2_(oda)_3_(phen)_4_]^2−^[Mn_2_(oda)(phen)_4_ (H_2_O)_2_]^2+^} (**7**)	2.79 ± 0.13****	26.12 ± 7.7
[Mn(oda)]⋅H_2_O (**8**)	> 100	> 100
1,10-phenathroline	33.17 ± 9.36**	20.88 ± 1.96
Cisplatin	40.10 ± 5.00	20.38 ± 2.27

IC_50_ (μM) represents the concentration at which there is a 50% reduction in metabolic activity in treated cells compared to untreated control cells. Mean ± SD (N = 3). * denotes *p* value < 0.05 compared to cisplatin; ** denotes *p* value < 0.01 compared to cisplatin; ***p value < 0.001 compared to cisplatin; ****p value < 0.0001 compared to cisplatin

The metal-phenanthroline complexes returned low IC_25_ and IC_50_ concentrations, exhibiting superior activity compared to the clinical therapeutic, cisplatin. Towards MCF-7 cells, the IC_50_ concentrations for all metal-phen complexes, excluding complex (**4**), were < 10 μM whereas the value for cisplatin was found to be 40.10 μM. Potential selectivity of the complexes was investigated by assessing the cytotoxicity towards non-cancerous MCF-12A cells. The cytotoxicity profiles yielded were largely similar, with the exception of complex **7**, containing an Mn(II) centre. The complex exhibited high potency towards the MCF-7 cells (IC_50_: 2.79 μM) (*p* < 0.0001), with an almost tenfold decrease in activity observed towards the MCF-12A cells (IC_50_: 26.12 μM). In contrast, cisplatin demonstrated increased activity towards MCF-12A cells than MCF-7 cells, with an IC_50_ of 20.38 μM. This indicates that not only does this complex demonstrate selectivity between cells lines but exhibits reduced toxicity in non-cancerous cells compared to cisplatin.

The activity of free 1,10-phenanthroline (Phen) was assessed in addition to the toxicity of the pre-cursor complexes (excluding phen ligand). Phen exhibited similar activity to cisplatin in both cell lines, being overall less potent than the metal-phen complexes. Both the Cu(II) and Mn(II) pre-cursors did not exhibit cytotoxicity below 100 μM, demonstrating the significance the phen-ligand had on the cytotoxicity of the complexes. The Ag(I) pre-cursor did exhibit activity; however, its toxicity was decreased by approximately twofold and 3.5-fold compared to the phen-containing complexes **4** and **5**, respectively.

### Detection of reactive oxygen species

The production of reactive oxygen species (ROS) was assessed to determine whether there may be a relationship between this stress stimuli and the cytotoxicity profiles of the complexes.

**Fig. 1:**
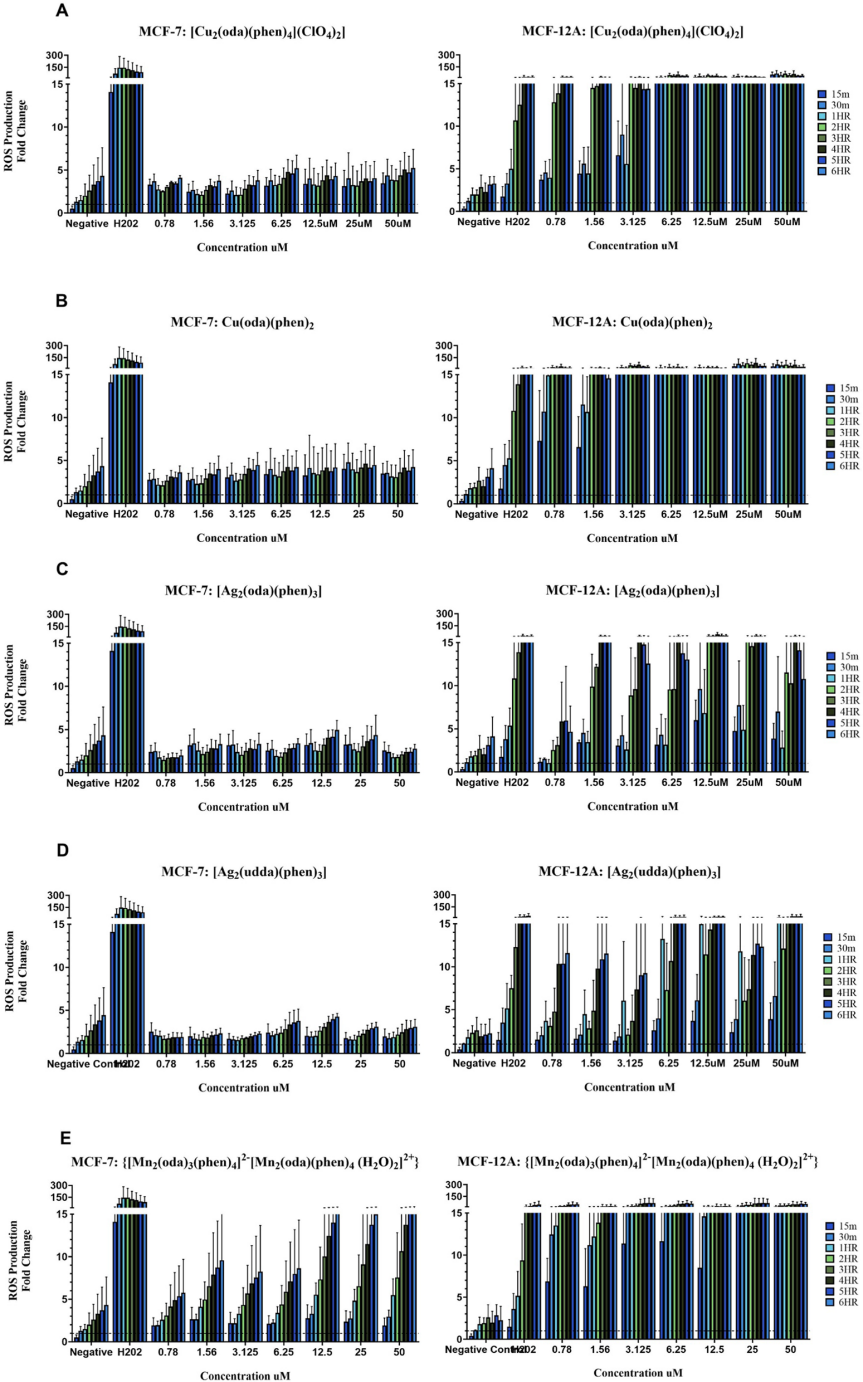
Detection of ROS production using H_2_DFCDA over a 6 h period following exposure to **A** [Cu_2_(oda)(phen)_4_](ClO_4_)_2_] (**1**). **B** Cu(oda)(phen)_2_ (**2**). **C** [Ag_2_(oda)(phen)_3_] (**4**). **D** [Ag_2_(udda)(phen)_3_] (**5**). **E** {[Mn_2_(oda)_3_(phen)_4_]^2−^[Mn_2_(oda)(phen)_4_(H_2_O)_2_]^2+^} (**7**). **F** Cisplatin. **G** 1,10-Phenanthroline

ROS production was evaluated in MFC-7 and MCF12A cells after exposure to complexes **1**,**2**,**4**,**5** and **7**, free 1,10-phenanthroline and cisplatin (Fig. 1). The cells were treated with a concentration range from 0.78 μM to 50  μM, and the response was measured over a 6-h period. This facilitated the evaluation of whether there was a time and/or concentration dependent response from the cells. Hydrogen peroxide (H_2_O_2_) was used as a positive control. ROS production is presented as fold-change compared to the negative control.

The Mn(II) complex (**7**) was found to be a potent inducer of ROS, eliciting the highest production in MCF-7 cells compared to the Cu(II) and Ag(I) complexes. This property was demonstrated previously by Kellett et al. (2011) and Slator et al. (2017. The MCF-12A cells were observed to have a markedly elevated production of ROS in response to the Mn(II) complex. An interesting result considering toxicity was reduced approximately tenfold in these cells respective to MCF-7 cells. Cancer cells have been seen to have impaired antioxidant defence systems compared to their healthy cell counterparts, often observed to have altered redox states making them more susceptible to stress induced by metal complexes (Gibellini et al. 2010). Making it conceivable that the MCF-12A cells are more adept at tolerating increased oxidative stress, protecting them to a certain extent. The Cu(II) complexes (**1** and **2**) elicited moderate production of ROS in MCF-7 cells, with the lowest production yielded in response to the Ag(I) complexes (**4** and **5**). The same trend in response to complex **7** was observed in the MCF-12A cells, with a much more pronounced production of ROS observed following exposure to the Cu(II) and Ag(I) complexes. The cytotoxicity of these complexes was observed to be very similar towards both cell lines, again suggesting the MCF-12A cells may have more robust antioxidant systems, allowing them to tolerate higher levels of ROS.

### Detection of apoptosis induction with flow cytometry

**Fig. 2:**
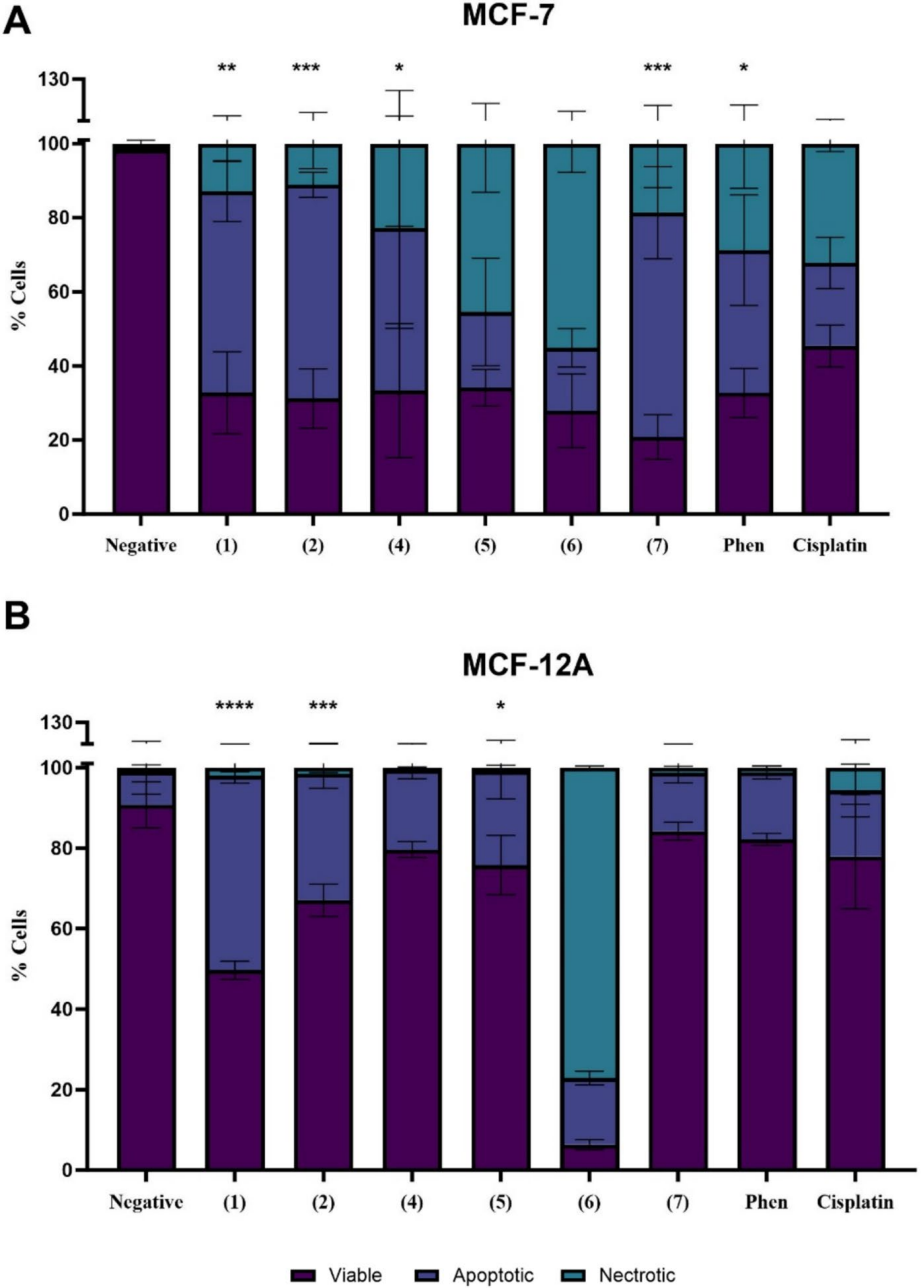
Apoptosis induction in MCF-7 and MCF-12A cells following exposure to IC_25_ concentrations of complexes **1**,**2**,**4**,**5**,**6** and **7**, 1,10-Phenanthroline and cisplatin. * denotes *p* value < 0.05; indicating significance compared to the negative control

The induction of apoptosis was analysed in MCF-7 and MCF-12A cells using the Annexin V and Propidium Iodide assay with flow cytometry. Apoptosis induction was evaluated following 24-h treatment with complexes **1**,**2**,**4**,**5**,**6**, and **7**, free 1,10-phenanthroline and cisplatin using the IC_25_ concentrations established from the cytotoxicity assessment. The assay allows for the differentiation of viable, apoptotic and necrotic cells based on the selective staining of Annexin V and PI. Annexin V binds to phosphatidylserines externalised to the outer leaflet of the plasma membrane during apoptosis; and PI is a membrane impermeable dye that binds to DNA of necrotic cells with compromised plasma membranes. Figure 2 shows the percentage population of cells (% cells) that are viable, apoptotic, and necrotic in MCF-7 and MCF-12A cells in response to each treatment.

Differential induction of apoptosis was observed in response to the complexes, depending on the metal centre, and with the responses also varying between the cell lines. The highest apoptotic population in both cell lines was observed in response to the Cu(II) complexes, with a higher population observed in the MCF-7 than MCF-12A cells. The apoptosis inducing ability of copper complexes is well reported in literature, with it widely accepted as one of the key mechanisms of action for these complexes (Santini et al. 2014; Abdolmaleki et al. 2024). There was a decrease in apoptotic populations in response to the Ag(I) complexes in MCF-7 cells, with high necrotic populations additionally observed, most notably in response to complex **6**. The response of MCF-12A cells in response to the complexes differed, with a significant decrease in apoptotic cells observed in response to complex **4**, and virtually no necrotic population. The same level of apoptosis was induced by complex **5**, however, no necrotic population was observed contrasting the significant population seen in MCF-7 cells. Complex **6** elicited a very similar response in both cell lines. The most pronounced apoptotic population was observed in MCF-7 cells following exposure to the Mn(II) complex (**7**), with a necrotic population also observed. This profile was highly contrasted in the MCF-12A cell line, with a significantly diminished apoptotic population and no necrosis observed.

The overall decrease in the levels of apoptosis induction in MCF-12A cells compared to their cancerous MCF-7 counterparts is of interest considering the toxicity profiles for the complexes were similar towards both cell lines, indicating alternative cell death pathways may be activated in the MCF-12A cells.

### Detection of caspase 3/7 expression with flow cytometry

**Fig. 3:**
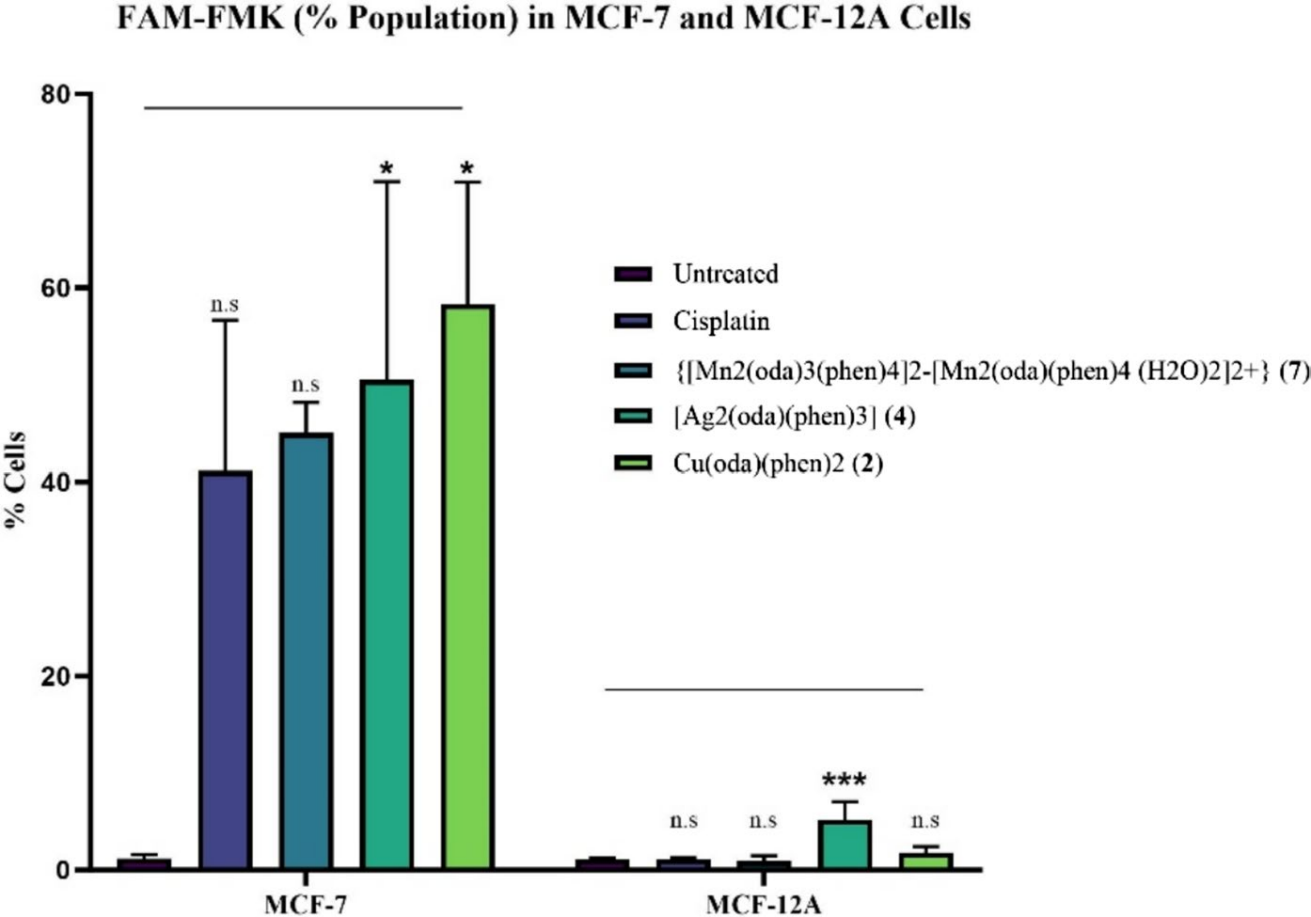
Caspase3/7 Expression in MCF-7 and MCF-12A cells following exposure to IC_25_ concentrations of Cu(oda)(phen)_2_ (**2**), [Ag_2_(oda)(phen)_3_] (**4**), {[Mn_2_(oda)_3_(phen)_4_]^2−^[Mn_2_(oda)(phen)_4_ (H_2_O)_2_]^2+^} (**7**) and cisplatin. * denotes *p* value < 0.05; indicating significance compared to the negative control

The expression of caspase-3/7 was analysed in MCF-7 and MCF-12A cells using fluorescent labelled inhibitors of caspase (FLICA): FAM-DEVD-FMK. Figure 3 displays the percentage of the population of cells (% cells) that were found to express caspase-3/7. Following the apoptosis induction study, it was of interest to determine whether the complexes were stimulating the executioner caspases triggering the irreversible proteolytic degradation of cells, culminating in cell death. Expression was assessed subsequent to 24-h treatment with complexes **2**, **4** and **7**, and cisplatin, using IC_25_ concentrations. These three complexes were selected based on their structural similarities, while incorporating alternative metal centres. This assay did not help distinguish whether caspase-3 or -7 specifically was expressed but established that either one of the executioner Caspases-3 or -7 was stimulated.

The highest expression of caspase-3/7 in MCF-7 cells was observed following exposure to the Cu(II) complex (**2**) (*p* value < 0.05). Followed by the Ag(I) (*p* value < 0.05) and Mn(II) complexes (**4** and **7**), with cisplatin eliciting the lowest expression levels. The Mn(II) complex having the lowest expression levels of the three complexes is of interest, considering in MCF-7 cells it elicited the highest apoptotic population. This suggests that while the pathway is stimulated by the complex, it may not be the executed, with an alternative cell death pathway taking place instead. Highly contrasting results were yielded between the MCF-7 and MCF-12A cells. The expression of caspase 3/7 was incredibly diminished for all complexes in the MCF-12A. The highest expression was observed for the Ag(I) complex (**4**) (*p* value < 0.001).

### Apoptosis-related gene expression

Upon establishing the ability of the metal-phen complexes to induce apoptosis to varying levels, it was of interest to determine the signalling cascade through which the pathway was progressing. Evaluation of apoptotic gene expression provides a more detailed insight into the mechanism of action of such complexes.

**Fig. 4:**
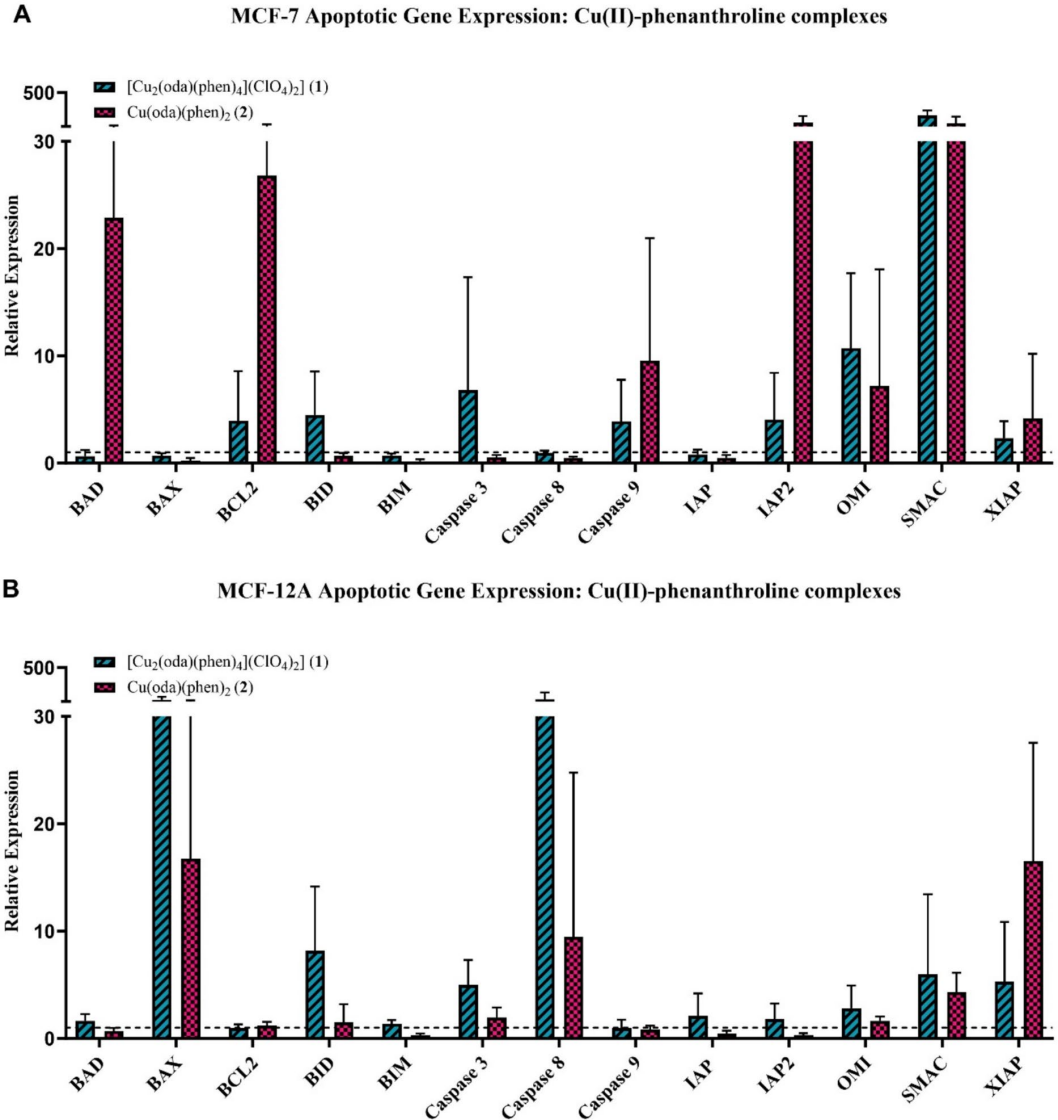
Gene expression in MCF-7 (**A**) and MCF-12A (**B**) cells following exposure to [Cu_2_(oda)(phen)_4_](ClO_4_)_2_] (**1**) and Cu(oda)(phen)_2_ (**2**) at IC_25_ concentrations

Apoptotic gene expression induced by complexes **1**,**2**,**4**,**5** and **7**, independent 1,10-phenanthroline and cisplatin was determined in MCF-7 and MCF-12A cells using qRT-PCR. The expression profiles (Fig. 4) display the log(2) transformed mean expression value for each gene target relative to the negative control. Expression values for each target were determined relative to the geometric means of reference genes. Gene expression is displayed as fold change relative to the reference genes. Each graph contains a threshold line, set at the value of one, this represents baseline expression levels of genes. Values above this line indicate upregulated genes, reflecting increased expression relative to the baseline. Conversely, values below the line signify downregulated genes, indicating a reduction in expression compared to the baseline.

Differential apoptotic responses were elicited by the metal-phen complexes in MCF-7 and MCF-12A cells, with notable differences in the regulatory methods of the cells. Cu(II) complexes (**1** and **2**) (Fig. 4A/B) upregulated expression of the gene encoding BH3-only protein *BID*, accompanied by expression of pro-apoptotic *BAD* and *BAX*. Higher expression of *BID* by MCF-12A cells suggests a higher initial apoptotic response. Deviations in anti-apoptotic gene expression was observed, with *BCL2* strongly expressed by MCF-7 cells compared to *XIAP* in MCF-12A cells, showing alternating pro-survival mechanisms between the cell lines. *IAP2* expression was markedly higher in MCF-7 cells but this was accompanied by high expression of mitochondrial *SMAC* and *OMI*, counteracting IAP activity. This expression pattern was also seen in MCF-12A cells but to a lesser extent. *Caspase-3* expression confirmed the execution of the pathway in both cell lines, with MCF-7 cells expressing initiator *caspase 9* whereas the MCF-12A cells expressed *caspase 8*. This implies the induction of the extrinsic pathway and subsequent activation of the intrinsic pathway via *Caspase-8* proteolytic cleavage of BID.

**Fig. 5:**
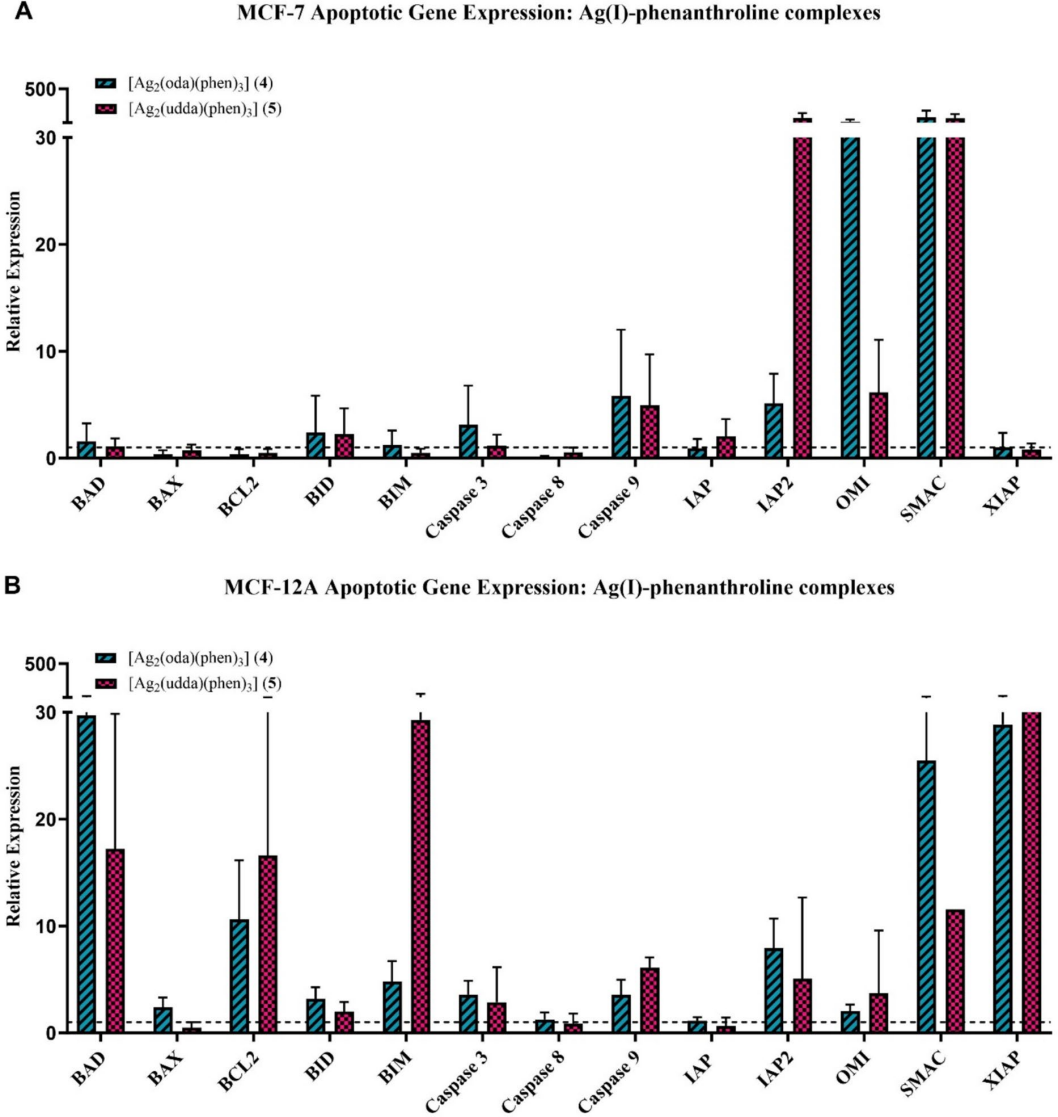
Gene expression in MCF-7 (**A**) and MCF-12A (**B**) cells following exposure to [Ag_2_(oda)(phen)_3_] (**4**) and [Ag_2_(udda)(phen)_3_] (**5**) at IC_25_ concentrations.

Exposure to the Ag(I) complexes (**4** and **5**) (Fig. 5A/B) elicited similar *BID* expression in both cell lines, but significantly elevated *BIM* and *BAD* expression was observed in MCF-12A cells. Similar to the Cu(II) complexes, this points to a higher initial apoptotic response in the non-cancerous cells. *BCL2* and *XIAP* expression was unique to the MCF-12A cells, potentially mitigating this elevated pro-apoptotic signalling and demonstrating a robust pro-survival mechanism in these cells. *IAP2* expression was similar between the cell lines, with its activity evidently sequestered by mitochondrial *SMAC/OMI* also seen to be upregulated. The expression of *caspase-9* and *caspase-3* again demonstrated the completion of the apoptotic cascade in both cell lines.

**Fig. 6:**
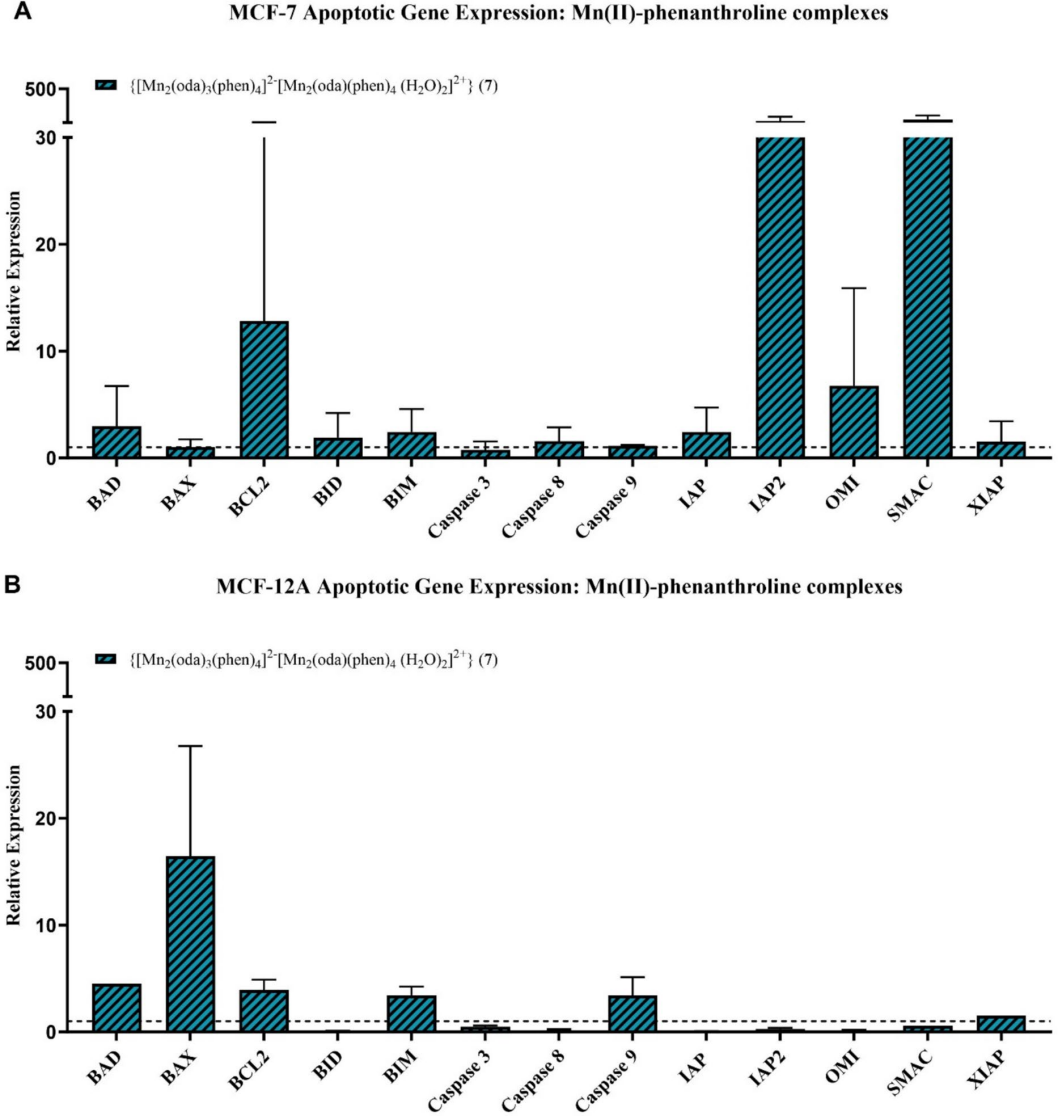
Gene expression in MCF-7 (**A**) and MCF-12A (**B**) cells following exposure to {[Mn_2_(oda)_3_(phen)_4_]_2_-[Mn_2_(oda)(phen)_4_ (H_2_O)_2_]^2+^} (**7**) at IC_25_ concentrations

Converse to the activity seen in response to the Cu(II) and Ag(I) complexes, exposure to the Mn(II) complex (Fig. 6A/B) elicited expression of both *BID* and *BIM* in MCF-7 cells with MCF-12A cells only shown to express *BIM*. Pro-apoptotic *BAD* was expressed in both cell lines, with *BAX* expression additionally observed in MCF-12A cells. No caspase activity was observed in the MCF-7 cells, suggesting the apoptotic cascade though activated, may not be executed in these cells. Initiator *caspase 9* was expressed by MCF-12A cells, but executioner *caspase-3* was not found to be expressed. Anti-apoptotic *BCL2* was expressed in both cell lines but upregulated more in MCF-7 cells. Pro-apoptotic *SMAC* and *OMI* were strongly expressed, with the activity of these mitochondrial genes reflected in the low-level expression of the inhibitors *IAP/XIAP*. The MCF-12A cells lacked expression of any apoptosis inhibitors and pro-apoptotic mitochondrial genes. Suggesting *BCL2* to be the main regulator of apoptosis for these cells.

**Fig. 7:**
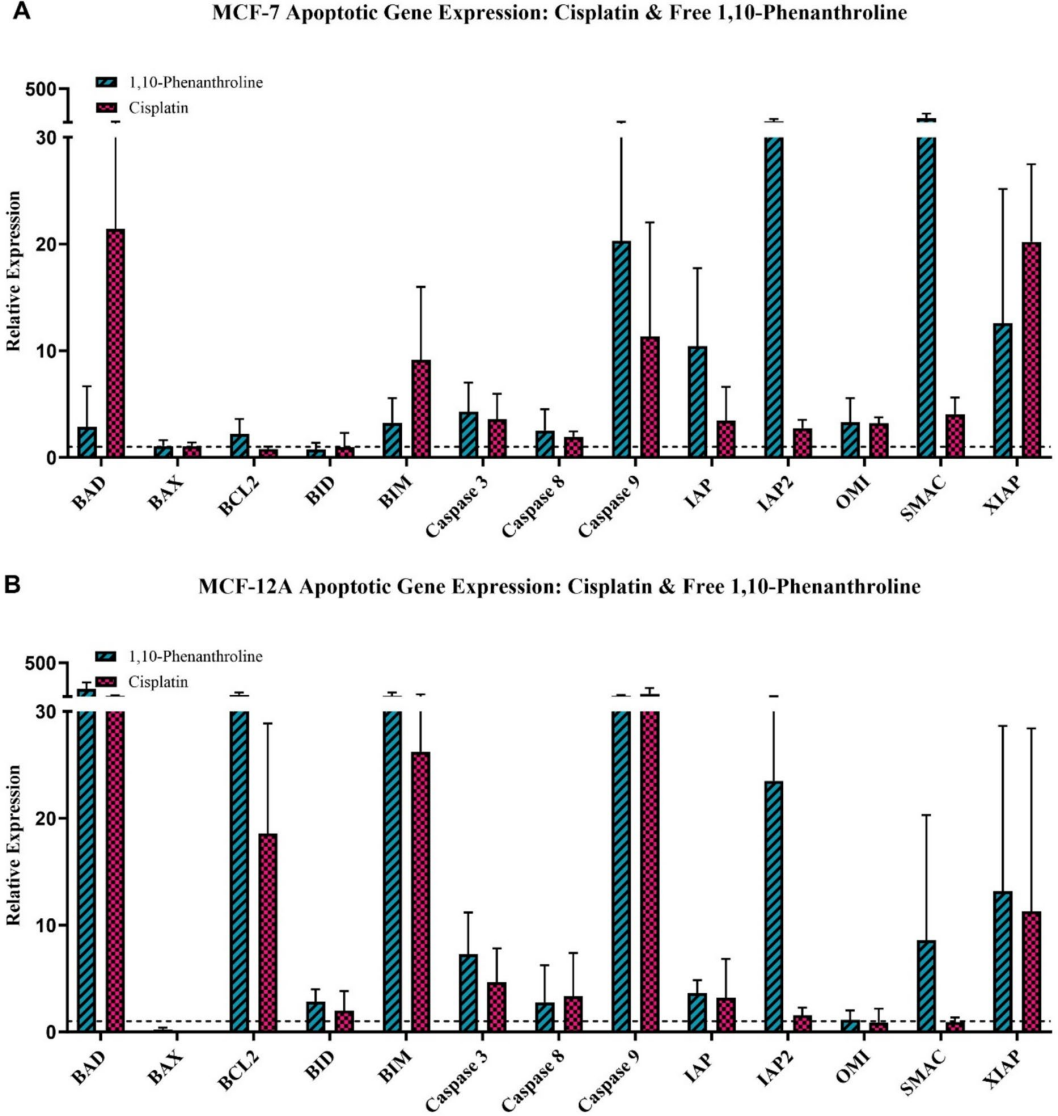
Gene expression in MCF-7 (**A**) and MCF-12A (**B**) cells following exposure to Cisplatin and free 1,10-phenanthroline at IC_25_ concentrations

The expression profiles of 1,10-Phenanthroline and cisplatin were largely similar (Fig. 7A/B), with the same behaviour observed in both cell lines as in response to the metal-phen complexes. Though pro-apoptotic *BIM* and *BAD* were upregulated in both cell lines, this expression was notably higher in the MCF-12A cells, accompanied by markedly elevated expression of anti-apoptotic *BCL2*. Both phen and cisplatin elicited a response from apoptosis inhibitors and pro-apoptotic mitochondrial genes in both cell lines, with all caspases seen to be activated in both cell lines.

The general trends observed from this gene expression study were that MCF-12As demonstrated higher pro-apoptotic gene expression in response to all the metal-phenanthroline complexes, accompanied by a higher anti-apoptotic regulation. Whereas the complexes elicited a lower pro-apoptotic response from MCF-7 cells, with these cells being less adept at counteracting the signals. This aligns with the cytotoxicity profiles established for the complexes as they were seen to have similar sensitivities, with the gene expression studies highlighting alternating response mechanisms from the cells.

## Discussion

The potential of Cu(II), Mn(II) and Ag(I) metal complexes with 1,10-Phenanthroline ligands as anti-cancer therapeutic agents was reported in numerous published studies including those by our research group and collaborators. Excellent in vitro cytotoxicity exceeding that of the current platinum-based chemotherapeutic cisplatin was reported, in addition to tumour-selectivity for some metal-phen complexes (Kellett et al. 2011; Thornton et al. 2016; Rochford et al. 2018). There were increased efforts to study how the metal-phen complexes exerted their effects on cells using cellular and molecular omics methodology (Rochford et al. 2018, 2020; O’Shaughnessy et al. 2023). All of these previous studies indicated differential cytotoxicity, and cellular mechanisms were contingent upon the metal centre present in the complex (Slator et al. 2017; Jakobsen et al. 2018; Rochford et al. 2020). Apoptosis is the most characterised regulated cellular death (RCD) pathway and is known to be induced by many external toxic agents including chemotherapeutic drugs such as cisplatin and its derivatives (carboplatin and oxaliplatin), NAMI-A, Ferroquine and Doxorubicin (Dasari and Tchounwou 2014; Jan and Chaudhry 2019; Anthony et al. 2020; Silvestri et al. 2021). We previously reported the significance of the apoptotic signalling cascade as a mechanism of cytotoxicity for Copper-Phen complexes such as Cu(o-phthalate)(phenanthroline)] (Slator et al. 2016) and Cu(II) phenanthroline-phenazine complexes (Rochford et al. 2020). It was therefore reasonable to assume that Apoptosis may be the dominant mode of cytotoxicity for our Copper(II) complex with dicarboxylate and 1,10-Phenanthroline ligand, and that it may also be a significant mechanistic mode of action for our Mn(II) and Ag(I) test complexes.

Herein, we have assessed the in vitro activity of the Cu(II), Mn(II) and Ag(I) complexes containing 1,10-phenanthroline ligands; and further explored the significance of apoptotic cell death as a mechanism of their cytotoxicity and therefore anticancer activity. Utilising MCF-7 (breast cancer) and MCF-12A (breast normal) cells provided a platform to observe potential differences in cellular responses and mechanistic action of tumour versus normal cells derived from the same tissue type. Incorporating cisplatin into the study enabled the comparison of the efficacy of the novel complexes to a clinical standard therapeutic.

In vitro cytotoxicity assessment established that the metal-phenanthroline complexes exhibit superior activity when compared to the clinical therapeutic, cisplatin. Overall, selectivity was not observed between the MCF-7 and MCF-12A cells by the metal-phen complexes, whereas cisplatin was observed to be more potent towards the MCF-12A than MCF-7 cells. The trend of cytotoxicity was as follows: Mn(II) (**7**) > Cu(II) (**1** and **2**) > Ag(I) (**4**, **5** and **6**) > 1,10-phenanthroline > cisplatin. Notably. the Mn(II) complex (**7**) was the sole complex observed to have selectivity between the cell lines, with an almost tenfold reduction in cytotoxicity observed. The notable cytotoxicity profiles of the Cu(II) and Mn(II) complexes correlate with previous studies conducted by our group and collaborators (Kellett et al. 2011). Our study established the complexes as potent inducers of apoptosis. It is clear from our data, that the generation of ROS is a significant contributor to their induction of this pathway. Combined with the previous observations that the complexes have DNA binding abilities, particularly with the knowledge that Cu(II) complexes can induce DNA double-strand breaks, it is evident that the metal-phen complexes demonstrate unique multi-modal activity, providing several avenues for their development as therapeutic agents (Kellett et al. 2011; Slator et al. 2017; Rochford et al. 2020). This present study further substantiates ROS induction as a major mechanism of action for Mn(II) complexes, with remarkable ROS inducing properties. In vivo studies of Mn(II) complexes have also highlighted the superior in vivo tolerance of the complexes compared to cisplatin (Kellett et al. 2011). In this context, the manganese-based complex **7** presents as a very interesting agent for continued exploration. With its significant cytotoxicity towards MCF-7 (cancerous) cells in tandem with its unique ROS stimulating properties compared to MCF12A cells, potential tumour –selectivity was evident for this breast tissue type but was not observed for the Copper(II) or Ag(I) complexes.

The mechanisms of action of the Ag(I) complexes within this study remain somewhat elusive. While Ag(I) complexes are well established as anti-microbial agents, their applications in cancer therapy have been reported to a significantly lower degree. A 2016 study by our group (Thornton et al. 2016) demonstrated the in vitro capabilities of a series of silver-phenanthroline complexes, highlighting their intercalative binding activity but without DNA damage. Ag(I) complexes have been recognised for their ability to elicit higher toxicity towards cancer cells than non-cancerous cells (Silva et al. 2020; Ota et al. 2021) This property makes them an attractive candidate for cancer therapy. The Ag(I) complexes analysed herein demonstrate impressive cytotoxicity, but not to the same extent as their Cu(II) and Mn(II) analogues. In tandem, though apoptotic responses were observed in response to these complexes, this response was reduced with a more pronounced necrotic response observed compared to its analogues. ROS induction was not determined to be a major mode of action for these complexes, highlighting the need for the continued exploration of the unique mechanisms underpinning the Ag(I) complexes. The complex interplay between cell death and pro-survival mechanisms was evidenced by the apoptotic gene expression study. Highlighting the intricate balance of signalling cascades necessary for cell death to occur.

We have therefore established the ability of Cu(II), Mn(II) and Ag(I) complexes incorporating bridging dicarboxylate and chelating 1,10-phenanthroline ligands to exhibit impressive cytotoxic activity in vitro. Their cytotoxicity can be attributed to the Apoptotic cellular death pathways for all complexes, in particular the Cu(II) and Mn(II) in tandem with ROS induction compared to the Ag(I) complexes. However, it was clear that while we established the role of apoptosis for the activity of our complexes, that this is one of many biological mechanisms responsible for their activity. Suggesting the metal-phen complexes are multi-modal in terms of their biological activity.

The use of the term “multi-modal” requires further clarification. In the context of this study, we used the term to reflect the combination of DNA-binding properties and ROS production observed here and in previous studies by our group. However, as highlighted by Gibson (Gibson 2019, 2021), the multi-modal activity of platinum(IV) complexes involves distinct and independently acting mechanisms, including DNA damage, signalling pathway modulation, HDAC inhibition, and immune target effects (e.g., IL-6 and NF-kB). These studies rely on speciation analysis to validate the independent biological effects of the released entities. Similarly, as broadly defined in Štarha and Trávníček’s 2019 review (Štarha and Trávníček 2019),, true multimodal behaviour requires demonstration of distinct, mechanistically independent biological effects resulting from speciation.

Without corresponding speciation studies, our complexes do not fully align with these previously published “multi-modal” studies. However, our ‘multi-modal’ terminology refers to the combined impact of DNA interactions, ROS generation and apoptosis induction by the complexes, rather than distinct, independently validated mechanisms of action with speciation analysis.

The results of our study therefore warrant continued exploration of the biological activity of this series of complexes. It is evident that there are alternative modes of regulated cell death to be explored, because our flow cytometry results were of particular interest. Several complexes elicited higher necrotic populations, and while these cells may simply be undergoing necrosis, it is also possible that an alternative form of cell death is occurring. This assumption arises from the identification of necrotic populations through propidium iodide (PI) staining, which intercalates with DNA upon the breakdown of the cell membrane. Notably, several regulated cell death (RCD) pathways, such as necroptosis, pyroptosis, ferroptosis, and autophagy can lead to membrane permeabilisation and subsequent PI uptake. Therefore, the presence of PI-positive cells suggests not only necrosis but also other forms of regulated cell death, warranting further investigation into the specific mechanisms and RCD pathways occurring (DiPeso et al. 2017; Ros et al. 2017; Ye et al. 2023; Hirata and Mishima 2024; Veeckmans et al. 2024).

## Supplementary Information

Below is the link to the electronic supplementary material.

Supplementary file1 (DOCX 265 KB)

## Data Availability

The authors confirm that the data supporting the findings of this study are available within the article. The raw data supporting the manuscript will be made available by the authors, without undue reservation, to any qualified researcher.

## References

[CR1] Abdolmaleki S, Aliabadi A, Khaksar S (2024) Unveiling the promising anticancer effect of copper-based compounds: a comprehensive review. J Cancer Res Clin Oncol. 10.1007/s00432-024-05641-5 38662225 10.1007/s00432-024-05641-5 PMC11045632

[CR2] Accorsi G et al (2009) 1,10-Phenanthrolines: versatile building blocks for luminescent molecules, materials and metal complexes. Chem Soc Rev 38(6):1690–1700. 10.1039/b806408n 19587962 10.1039/b806408n

[CR3] Ahmed M et al (2019) Synthesis and antimicrobial activity of a phenanthroline-isoniazid hybrid ligand and its Ag^+^ and Mn^2+^ complexes. BioMetals 32(4):671–682. 10.1007/s10534-019-00204-5 31230149 10.1007/s10534-019-00204-5

[CR4] Al-Aamri HM et al (2021) Intrinsic and extrinsic apoptosis responses in leukaemia cells following daunorubicin treatment. BMC Cancer. 10.1186/s12885-021-08167-y 33879127 10.1186/s12885-021-08167-y PMC8059319

[CR5] Alreja P, Kaur N (2016) Recent advances in 1,10-phenanthroline ligands for chemosensing of cations and anions. RSC Adv. 10.1039/c6ra00150e

[CR6] Anthony EJ et al (2020) Metallodrugs are unique: opportunities and challenges of discovery and development. Chem Sci 11(48):12888–12917. 10.1039/d0sc04082g 34123239 10.1039/d0sc04082g PMC8163330

[CR7] Baig S et al (2016) Potential of apoptotic pathway-targeted cancer therapeutic research: where do we stand. Cell Death Dis. 10.1038/cddis.2015.275 26775709 10.1038/cddis.2015.275 PMC4816162

[CR100] Casey, M. T., McCann, M., Devereux, M., Curran, M., Cardin, C., Convery, M., et al. (1994). Synthesis and structure of the Mn2 (II, II) complex salt [Mn2(oda)(phen)4(H2O)2] [Mn2(oda)2(phen)4] (odaH2= octanedioic acid): a catalyst for H2O2 disproportionation. J. Chem. Soc. Chem. Commun. 22, 2643–2645. 10.1039/C39940002643

[CR8] Dasari S, Bernard Tchounwou P (2014) Cisplatin in cancer therapy: molecular mechanisms of action. Eur J Pharm. 10.1016/j.ejphar.2014.07.025 10.1016/j.ejphar.2014.07.025 PMC4146684 25058905

[CR10] DiPeso L et al (2017) Cell death and cell lysis are separable events during pyroptosis. Cell Death Discov. 10.1038/cddiscovery.2017.70 29147575 10.1038/cddiscovery.2017.70 PMC5682879

[CR11] Đurić S et al (2020) Silver(I) complexes with 1,10-phenanthroline-based ligands: the influence of epoxide function on the complex structure and biological activity. Inorg Chim Acta. 10.1016/j.ica.2019.119357

[CR12] Gałczyńska K, Drulis-Kawa Z, Arabski M (2020) Antitumor activity of Pt(II), Ru(III) and Cu(II) complexes. Molecules. 10.3390/molecules25153492 32751963 10.3390/molecules25153492 PMC7435640

[CR13] Galluzzi L et al (2018) ‘Molecular mechanisms of cell death: recommendations of the nomenclature committee on cell death 2018. Cell Death Differ. 10.1038/s41418-017-0012-4 29362479 10.1038/s41418-017-0012-4 PMC5864239

[CR14] Gandra RM et al (2017) Antifungal potential of copper(II), manganese(II) and silver(I) 1,10-phenanthroline chelates against multidrug-resistant fungal species forming the *Candida haemulonii* complex: impact on the planktonic and biofilm lifestyles. Front Microbiol. 10.3389/fmicb.2017.01257 28744261 10.3389/fmicb.2017.01257 PMC5504357

[CR15] Gandra RM et al (2020) In vivo activity of copper(II), manganese(II), and silver(I) 1,10-phenanthroline chelates against *Candida haemulonii* using the *Galleria mellonella* model. Front Microbiol. 10.3389/fmicb.2020.00470 32265890 10.3389/fmicb.2020.00470 PMC7105610

[CR16] Gibellini L et al (2010) Interfering with ROS metabolism in cancer cells: the potential role of quercetin. Cancers. 10.3390/cancers2021288 24281116 10.3390/cancers2021288 PMC3835130

[CR17] Gibson D (2019) ‘Multi-action Pt(IV) anticancer agents; do we understand how they work? J Inorg Biochem. 10.1016/j.jinorgbio.2018.11.008 30471522 10.1016/j.jinorgbio.2018.11.008

[CR18] Gibson D (2021) Platinum(IV) anticancer agents; are we en route to the holy grail or to a dead end? J Inorg Biochem. 10.1016/j.jinorgbio.2020.111353 33477089 10.1016/j.jinorgbio.2020.111353

[CR19] Granato MQ et al (2017) 1,10-phenanthroline-5,6-dione-based compounds are effective in disturbing crucial physiological events of *Phialophora verrucosa*. Front Microbiol. 10.3389/fmicb.2017.00076 28194139 10.3389/fmicb.2017.00076 PMC5276843

[CR20] Granato MQ et al (2021) ‘Silver(I) and copper(II) complexes of 1,10-phenanthroline-5,6-dione against *Phialophora verrucosa*: a focus on the interaction with human macrophages and *Galleria mellonella* Larvae. Front Microbiol. 10.3389/fmicb.2021.641258 34025603 10.3389/fmicb.2021.641258 PMC8138666

[CR21] Green DR (2022) The death receptor pathway of apoptosis death receptors are a subset of the tumor necrosis factor receptor family. Available at: http://cshperspectives.cshlp.org/

[CR22] Hirata Y, Mishima E (2024) Membrane dynamics and cation handling in ferroptosis. Physiology. 10.1152/physiol.00029.2023 38193763 10.1152/physiol.00029.2023 PMC11283900

[CR23] Jakobsen V et al (2018) Tetrameric and polymeric silver complexes of the omeprazole scaffold; synthesis, structure, in vitro and in vivo antimicrobial activities and DNA interaction. J Inorg Biochem 186:317–328. 10.1016/j.jinorgbio.2018.05.018 30025225 10.1016/j.jinorgbio.2018.05.018

[CR24] Jan R, Chaudhry GES (2019) Understanding apoptosis and apoptotic pathways targeted cancer therapeutics. Adv Pharm Bull. 10.15171/apb.2019.024 31380246 10.15171/apb.2019.024 PMC6664112

[CR25] Kellett A et al (2011) Water-soluble bis(1,10-phenanthroline) octanedioate Cu^2+^ and Mn^2+^ complexes with unprecedented nano and picomolar in vitro cytotoxicity: promising leads for chemotherapeutic drug development. MedChemComm 2(7):579–584. 10.1039/c0md00266f

[CR26] Kellett A et al (2012) Radical-induced DNA damage by cytotoxic square-planar copper(II) complexes incorporating o-phthalate and 1,10-phenanthroline or 2,2′-dipyridyl. Free Radical Biol Med 53(3):564–576. 10.1016/j.freeradbiomed.2012.05.034 22659117 10.1016/j.freeradbiomed.2012.05.034

[CR27] Livak KJ, Schmittgen TD (2001) Analysis of relative gene expression data using real-time quantitative PCR and the 2^-ΔΔCT^ method. Methods 25(4):402–408. 10.1006/meth.2001.1262 11846609 10.1006/meth.2001.1262

[CR28] Mateâ JM, Saâ Nchez-Jimeâ Nez FM (2000) Role of reactive oxygen species in apoptosis: implications for cancer therapy. Int J Biochem Cell Biol. www.elsevier.com/locate/ijbcb 10.1016/s1357-2725(99)00088-6 10687951

[CR29] Mccann M et al (1995) COPPER(I1) COMPLEXES OF HEPTANEDIOIC ACID (hdaHz) AND OCTANEDIOIC ACID (odaH,): X-RAY CRYSTAL STRUCTURES OF [Cu(q*-hda)(phen),] * 11.73&o AND [Cu(q*-oda)(phen),]-12H20 (phen = l,lO-PHENANTHROLINE), Polyhedron

[CR30] McCarron P et al (2018) Unprecedented in vitro antitubercular activitiy of manganese(II) complexes containing 1,10-phenanthroline and dicarboxylate ligands: increased activity, superior selectivity, and lower toxicity in comparison to their copper(II) analogs. Front Microbiol. 10.3389/fmicb.2018.01432 30013535 10.3389/fmicb.2018.01432 PMC6036174

[CR9] Michael Devereux et al (1994) Binuclear and polymeric copper(II) dicarboxylate complexes: syntheses and crystal structures of [Cu (pda)(Phen) ](ClO ) ?5H O?C H OH, 2 4 4 2 2 2 5 [Cu (oda)(Phen) ](ClO ) ?2.67H O?C H OH and 2 4 4 2 2 2 5 h[Cu (pda) (NH ) (H O) ]?4H Oj (odaH 5octanedioic acid; 2 2 3 4 2 2 2 n 2 pdaH 5pentanedioic acid; Phen51,10-phenanthroline)

[CR31] Molinaro C et al (2020) Copper complexes as anticancer agents targeting topoisomerases i and ii. Cancers. 10.3390/cancers12102863 33027952 10.3390/cancers12102863 PMC7601307

[CR32] Nunes P et al (2020) Copper complexes with 1,10-phenanthroline derivatives: underlying factors affecting their cytotoxicity. Inorg Chem 59(13):9116–9134. 10.1021/acs.inorgchem.0c00925 32578983 10.1021/acs.inorgchem.0c00925

[CR33] O’Shaughnessy M et al (2023) Antibacterial activity of metal–phenanthroline complexes against multidrug-resistant Irish clinical isolates: a whole genome sequencing approach. J Biol Inorg Chem 28(2):153–171. 10.1007/s00775-022-01979-8 36484826 10.1007/s00775-022-01979-8 PMC9734640

[CR34] Ocker M, Höpfner M (2012) Apoptosis-modulating drugs for improved cancer therapy. Eur Surg Res. 10.1159/000336875 22538523 10.1159/000336875

[CR35] Ota A et al (2021) The selective cytotoxicity of silver thiosulfate, a silver complex, on MCF-7 breast cancer cells through ROS-induced cell death. Pharmacol Rep 73(3):847–857. 10.1007/s43440-021-00260-0 33864630 10.1007/s43440-021-00260-0 PMC8180477

[CR36] Redza-Dutordoir M, Averill-Bates DA (2016) Activation of apoptosis signalling pathways by reactive oxygen species. Biochimica et Biophysica Acta. 10.1016/j.bbamcr.2016.09.012 27646922 10.1016/j.bbamcr.2016.09.012

[CR37] Rochford G et al (2018) In-vivo evaluation of the response of *Galleria mellonella* larvae to novel copper(II) phenanthroline-phenazine complexes. J Inorg Biochem 186:135–146. 10.1016/j.jinorgbio.2018.05.020 29906780 10.1016/j.jinorgbio.2018.05.020

[CR38] Rochford G et al (2020) Cu(ii) phenanthroline-phenazine complexes dysregulate mitochondrial function and stimulate apoptosis. Metallomics 12(1):65–78. 10.1039/c9mt00187e 31720645 10.1039/c9mt00187e

[CR39] Ros U et al (2017) Necroptosis execution is mediated by plasma membrane nanopores independent of calcium. Cell Rep 19(1):175–187. 10.1016/j.celrep.2017.03.024 28380356 10.1016/j.celrep.2017.03.024 PMC5465952

[CR40] Roy S, Nicholson DW (2000) Commentary cross-talk in cell death signalling. J Exp Med. Available at: http://www.jem.org/cgi/content/full/192/8/F21 11034597

[CR41] Sammes PG, Yahioglu G (1994) 1,10-Phenanthroline: a versatile ligand. Chem Soc Rev 23:327–334. 10.1039/CS9942300327

[CR42] Santini C et al (2014) Advances in copper complexes as anticancer agents. Cheml Rev. 10.1021/cr400135x 10.1021/cr400135x 24102434

[CR43] Santos ALS et al (2012) Antimicrobial action of chelating agents: repercussions on the microorganism development, virulence and pathogenesis. Curr Med Chem 19:2715–2737 22455582 10.2174/092986712800609788

[CR44] Silva DES et al (2020) Cytotoxic and apoptotic effects of ternary silver(i) complexes bearing 2-formylpyridine thiosemicarbazones and 1,10-phenanthroline. Dalton Trans 49(16):5264–5275. 10.1039/d0dt00253d 32242564 10.1039/d0dt00253d

[CR45] Silvestri S et al (2021) Evaluation of anticancer role of a novel ruthenium(II)-based compound compared with NAMI-A and cisplatin in impairing mitochondrial functionality and promoting oxidative stress in triple negative breast cancer models. Mitochondrion 56:25–34. 10.1016/j.mito.2020.11.004 33220497 10.1016/j.mito.2020.11.004

[CR46] Slator C et al (2016) Exhibits unique superoxide-mediated NCI-60 chemotherapeutic action through genomic DNA damage and mitochondrial dysfunction. ACS Chem Biol 11(1):159–171. 10.1021/acschembio.5b00513 26488846 10.1021/acschembio.5b00513

[CR47] Slator C et al (2017) Triggering autophagic cell death with a di-manganese(II) developmental therapeutic. Redox Biol 12:150–161. 10.1016/j.redox.2017.01.024 28236767 10.1016/j.redox.2017.01.024 PMC5328722

[CR49] Štarha P, Trávníček Z (2019) Non-platinum complexes containing releasable biologically active ligands. Coord Chem Rev. 10.1016/j.ccr.2019.06.001

[CR50] Tang D et al (2019) The molecular machinery of regulated cell death. Cell Res. 10.1038/s41422-019-0164-5 30948788 10.1038/s41422-019-0164-5 PMC6796845

[CR51] Thornton L et al (2016) ‘Water-soluble and photo-stable silver(I) dicarboxylate complexes containing 1,10-phenanthroline ligands: antimicrobial and anticancer chemotherapeutic potential, DNA interactions and antioxidant activity. J Inorg Biochem 159:120–132. 10.1016/j.jinorgbio.2016.02.024 26986979 10.1016/j.jinorgbio.2016.02.024

[CR52] Vandesompele J et al (2002) Accurate normalization of real-time quantitative RT-PCR data by geometric averaging of multiple internal control genes. Available at: http://genomebiology.com/2002/3/7/research/0034.1Correspondence:.rankSpeleman 10.1186/gb-2002-3-7-research0034 PMC126239 12184808

[CR53] Veeckmans G, Van San E, Vanden Berghe T (2024) A guide to ferroptosis, the biological rust of cellular membranes. FEBS J 291(13):2767–2783. 10.1111/febs.16993 37935445 10.1111/febs.16993

[CR54] Ventura RF et al (2020) Antimicrobial action of 1,10-phenanthroline-based compounds on carbapenemase-producing *Acinetobacter baumannii* clinical strains: efficacy against planktonic- and biofilm-growing cells. Braz J Microbiol 51(4):1703–1710. 10.1007/s42770-020-00351-9 32737867 10.1007/s42770-020-00351-9 PMC7688763

[CR55] Vianez Peregrino I et al (2021) Antibacterial activity and carbapenem re-sensitizing ability of 1,10-phenanthroline-5,6-dione and its metal complexes against KPC-producing *Klebsiella pneumoniae* clinical strains. Lett Appl Microbiol 73(2):139–148. 10.1111/lam.13485 33843058 10.1111/lam.13485

[CR56] Viganor L et al (2016) The antibacterial activity of metal complexes containing 1,10- phenanthroline: potential as alternative therapeutics in the era of antibiotic resistance. Curr Top Med Chem 17(11):1280–1302. 10.2174/1568026616666161003143333 10.2174/1568026616666161003143333 27697043

[CR57] Villalpando-Rodriguez GE, Gibson SB (2021) Reactive oxygen species (ROS) regulates different types of cell death by acting as a rheostat. Oxid Med Cell Longevity. 10.1155/2021/9912436 10.1155/2021/9912436 PMC8380163 34426760

[CR58] Ye K, Chen Z, Xu Y (2023) The double-edged functions of necroptosis. Cell Death Dis. 10.1038/s41419-023-05691-6 36849530 10.1038/s41419-023-05691-6 PMC9969390

