# Supplementary material for: Apoptotic cell death induced by copper (II), manganese (II) and silver (I) complexes containing bridging dicarboxylate and 1,10-phenanthroline ligands: one of the multi-modes of anticancer activity?
Source: Biometals. 2025 Mar 17;38(3):785–805. doi: 10.1007/s10534-025-00676-8 (PMC12119673; doi:10.1007/s10534-025-00676-8)
Supplement: Supplementary file 1 — Supplementary file1 (DOCX 265 KB) [file 10534_2025_676_MOESM1_ESM.docx]

**Supplementary Table 1** Chemical formulae and structures of active complexes

| **Code** | **Formula** | **Structure** |
| --- | --- | --- |
| **Complex 1** | [Cu_2_(oda)(phen)_4_](ClO_4_)_2_ | 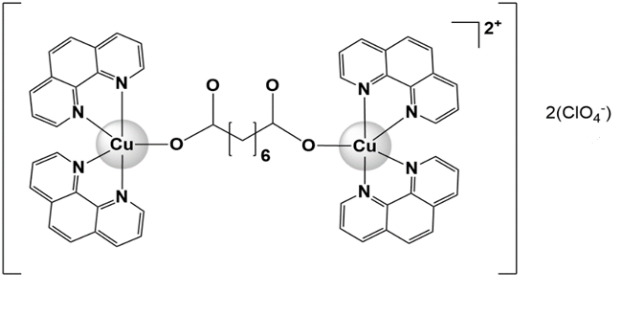 |
| **Complex 2** | [Cu(oda)(phen)_2_] | 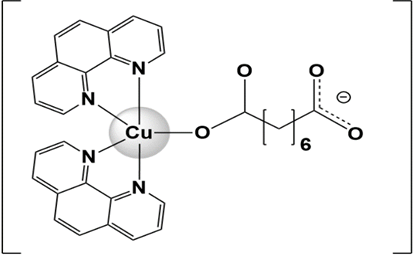 |
| **Complex 4** | [Ag_2_(oda)(phen)_3_] | **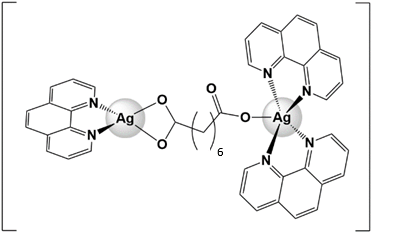** |
| **Complex 5** | [Ag_2_(udda)(phen)_3_] | **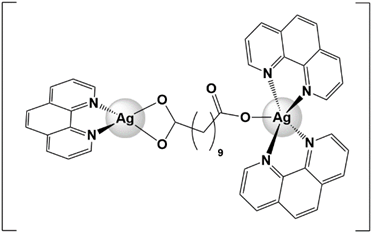** |
| **Complex 6** | [Ag_2_(oda)] | **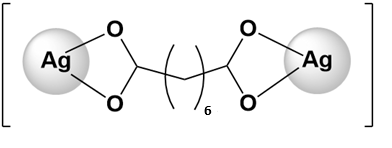** |
| **Complex 7** | {[Mn_2_(oda)_3_(phen)_4_]^2-^[Mn_2_(oda)(phen)_4_ (H_2_O)_2_]^2+^} | 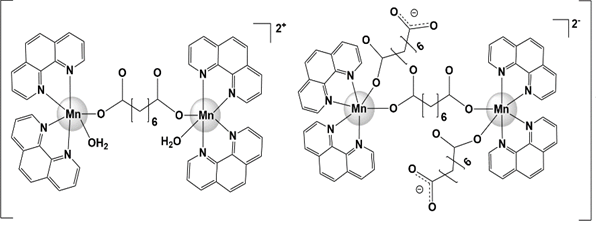 |

**Supplementary Table 2** IC_25_ values for MCF-7 and MCF-12A cells following 24-hour exposure to metal-phenanthroline complexes, 1,10-phenanthroline and cisplatin; calculated per one metal centre.

| Complex | MCF-7 | MCF-12A |
| --- | --- | --- |
| [Cu2(oda)(phen)4](ClO4)2] (**1**) ​ | 3.54 ± 0.28** ​ | 9.72 ± 3.82 |
| [Cu(oda)(phen)2] (**2**) ​ | 2.04 ± 0.24* ​ | 0.68 ± 0.13 |
| [Cu2(oda)2] (**3**) ​ | >100 ​ | >100 ​ |
| [Ag2(oda)(phen)3] (**4**) ​ | 11.04 ± 1.78 ​ | 9.82 ± 0.28 |
| [Ag2(udda)(phen)3] (**5**) ​ | 11.68 ± 0.24 ​ | 11.04 ± 1.0 |
| [Ag2(oda)] (**6**) ​ | 27.80 ± 5.86 | 12.86 ± 9.80 |
| {[Mn2(oda)3(phen)4]^2-^[Mn2(oda)(phen)4 (H2O)2]^2+^} (**7**) ​ | 2.80 ± 0.44** ​ | 1.52 ± 1.56 |
| [Mn(oda)].H2O  (**8**) | >100 ​ | >100 ​ |
| 1,10-phenathroline | 9.02 ± 4.26 ​ | 11.14 ± 4.39 |
| Cisplatin ​ | 11.77 ± 6.6 ​ | 7.84 ± 2.47 |

IC_25_ (μM) represents the concentration at which there is a 25% reduction in metabolic activity in treated cell compared to untreated control cells. Mean ± S.D. (N=3). * denotes 𝘱 value < 0.05 compared to cisplatin; ** denotes 𝘱 value < 0.01 compared to cisplatin; *** 𝘱 value < 0.001 compared to cisplatin; **** 𝘱 value < 0.0001 compared to cisplatin

**Supplementary Table 3** IC_50_ values for MCF-7 and MCF-12A cells following 24-hour exposure to metal-phenanthroline complexes, 1,10-phenanthroline and cisplatin; calculated per one metal centre.

| Complex | MCF-7 | MCF-12A |
| --- | --- | --- |
| [Cu2(oda)(phen)4](ClO4)2] (**1**) ​ | 9.52 ± 0.60**** ​ | 12.28 ± 0.74* |
| [Cu(oda)(phen)2] (**2**) ​ | 6.72 ± 0.60**** ​ | 1.65 ± 0.13** ​ |
| [Cu2(oda)2] (**3**) ​ | >100 ​ | >100 ​ |
| [Ag2(oda)(phen)3] (**4**) ​ | 27.06 ± 2.96**** ​ | 12.24 ± 0.66​* |
| [Ag2(udda)(phen)3] (**5**) ​ | 15.36 ± 0.18**** ​ | 13.56 ± 1.00​* |
| [Ag2(oda)] (**6**) ​ | 53.54 ± 14.28* ​ | 28.72 ± 6.24​ |
| {[Mn2(oda)3(phen)4]^2-^[Mn2(oda)(phen)4 (H2O)2]2+} (**7**) ​ | 11.16 ± 0.52**** ​ | 104.48 ± 30.8​ |
| [Mn(oda)].H2O  (**8**) | >100 ​ | >100 ​ |
| 1,10-phenathroline | 33.17 ± 9.36** ​ | 20.88 ± 1.96​ |
| Cisplatin ​ | 40.10 ± 5.00 ​ | 20.38 ± 2.27​ |

IC_50_ (μM) represents the concentration at which there is a 50% reduction in metabolic activity in treated cell compared to untreated control cells. Mean ± S.D. (N=3). * denotes 𝘱 value < 0.05 compared to cisplatin; ** denotes 𝘱 value < 0.01 compared to cisplatin; *** 𝘱 value < 0.001 compared to cisplatin; **** 𝘱 value < 0.0001 compared to cisplatin
